# Incidental inequity

**DOI:** 10.1038/s41431-018-0101-y

**Published:** 2018-02-15

**Authors:** Kristen J. Nowak, Alicia Bauskis, Hugh J. Dawkins, Gareth Baynam

**Affiliations:** 1 Department of Health, Office of Population Health Genomics, Public and Aboriginal Health Division, Government of Western Australia, East Perth, WA Australia; 2 School of Biomedical Sciences, Faculty of Health and Medical Sciences, University of Western Australia, Crawley, WA Australia; 3 Harry Perkins Institute of Medical Research, QEII Medical Centre, Nedlands; Centre for Medical Research, University of Western Australia, Crawley, WA Australia; 4 Centre for Population Health Research, Curtin Health Innovation Research Institute, Curtin University of Technology, Bentley, WA Australia; 5 Centre for Comparative Genomics, Murdoch University, Murdoch, WA Australia; 6 Department of Health, Genetic Services of Western Australia, Government of Western Australia, Subiaco, WA Australia; 7 School of Paediatrics and Child Health, University of Western Australia, Crawley, WA Australia; 8 Institute for Immunology and Infectious Diseases, Murdoch University, Murdoch, WA Australia; 9 Telethon Kids Institute, University of Western Australia, Subiaco, WA Australia; 10 Western Australian Register of Developmental Anomalies, King Edward Memorial Hospital, Subiaco, WA Australia; 11 School of Spatial Sciences, Curtin University, Bentley, WA Australia

Incidental findings (also known as unsolicited or secondary findings) in genomics are genetic variants that are detected in an individual, yet are unrelated to the current diagnostic question and the phenotype (associated features) of the disorder for which that person is being investigated [1]. Reporting incidental genomic findings requires various considerations. One of these is that 'the clinical validity and utility of variants should be known' [2], which is especially pertinent for those populations with a paucity of genomic reference data. Indeed, the need for great caution has been vividly highlighted by reversal of the predicted consequence of variants detected on investigation of cardiomyopathy in African Americans. Specifically, variants previously reported as pathogenic (affecting function, disease-causing) were reclassified as non-pathogenic (not affecting function, benign), when results were reviewed in the light of ethnic-specific reference data [3]. Our concern is that there is a dearth of specific genomic reference ranges for various populations, including many Indigenous populations and that this limits the potential for them to benefit from genomic medicine.

The likelihood of requiring retraction of false genetic assertions, for or against a variant contributing to disease, is greatest in settings of the lowest pre-test probability of disease causation. In this instance, low pre-test probability is when a low likelihood exists prior to the diagnostic test result being known of a patient having a variant affecting function in a gene-unrelated (i.e. incidental) to the phenotype under investigation. Reclassification of clinical genetic findings (especially those identified incidentally) has far-reaching implications. These involve living with unnecessary medical interventions, which could involve surgery such as mastectomy, colectomy, or implant of a cardiac defibrillator based on an erroneous inherited breast cancer, colon cancer or arrhythmia predisposition test result, respectively. Additional implications include, but are not limited to unnecessary investigations, false restriction of employment and insurance, family planning decisions and overall patient anxiety. Furthermore, an individual may test negative for a variant that was erroneously classified as affecting function and incorrectly attributed as the cause of a known disease in their family. In such a case, where the culprit genetic variant remains unidentified, there will be false reassurance of the person’s risk of otherwise avoidable disease complications (possibly including death). These effects can be amplified through multiple family members and the health system.

Indigenous populations around the world experience increased disability as well as reduced health, quality of life and lifespan compared to their non-Indigenous equivalents [4]. Therefore, there is an immense need to close this health inequity gap and to ensure that Indigenous populations are able to gain advantage from new genomics knowledge and application. This requires considered, cohesive, culturally sensitive and community-engaged initiatives. However, the present unavailability of genomic databases for indigenous populations has the possibility to hamper their benefit from genomic medicine. In an Australian context, we are at a transition stage of increasing genomic knowledge and its clinical implementation for Indigenous Australians. We are at a delicate turning point that has a historical context of mistrust of genetic and genomic investigations [5]. For Indigenous populations such as Australia’s, there is compelling need to build trust to increase engagement in genomic medicine and to thereby result in improved health for these individuals.

We endorse recommendations that presently in genomic medicine, an approach targeted to the gene/s relevant to the condition being investigated in a patient is preferred, e.g. [6]. Moreover, should incidental findings be uncovered in the context of a diagnostic approach targeted to the phenotype, extra weighting should be given not to report them until the level of certainty meets the required quality thresholds for implementation to ensure best clinical care. We emphasise that this is especially pertinent until relevant, population-specific and representative genomic databases are available. The absence of such databases result in inequity for the application of genomic medicine to Indigenous and other unrepresented ethnic populations. Therefore, it is crucial that genomic information is compiled for peoples currently without reference data.
